# Investigating the relationship between serum uric acid to high‐density lipoprotein ratio and metabolic syndrome

**DOI:** 10.1002/edm2.311

**Published:** 2021-10-27

**Authors:** Farzaneh Yazdi, Mohammad Hassan Baghaei, Amir Baniasad, Ahmad Naghibzadeh‐Tahami, Hamid Najafipour, Mohammad Hossein Gozashti

**Affiliations:** ^1^ Neuroscience Research Center Institute of Neuropharmacology Kerman University of Medical Sciences Kerman Iran; ^2^ Gastroenterology and Hepathology Research Center Institute of Basic and Clinical Physiology Sciences Kerman University of Medical Sciences Kerman Iran; ^3^ Endocrinology and Metabolism Research Center Institute of Basic and Clinical Physiology Science Kerman University of Medical Sciences Kerman Iran; ^4^ Physiology Research Center Institute of Basic and Clinical Physiology Sciences Kerman University of Medical Sciences Kerman Iran; ^5^ Cardiovascular Research Center Institute of Basic and Clinical Physiology Sciences Kerman University of Medical Sciences Kerman Iran; ^6^ Endocrinology and Metabolism Research Center Institute of Basic and Clinical Physiology Sciences Kerman University of Medical Sciences Kerman Iran

**Keywords:** HDL, metabolic syndrome, UHR, uric acid

## Abstract

**Aims:**

This study aimed to determine a parameter to more easily diagnose metabolic syndrome and predict its probability of occurrence in high‐risk individuals.

**Methods:**

In this cross‐sectional study, data related to the study population in the Kerman Coronary Artery Disease Risk Factor Study (KERCADRS) were examined. Subjects were divided into two groups with and without metabolic syndrome, and the relevant factors such as the ratios of uric acid to high‐density lipoprotein (HDL) (UHR) in these two groups were compared, and the best cut‐off point was determined.

**Results:**

Data related to 817 people including 96 people with metabolic syndrome and 721 people without metabolic syndrome were analysed. The mean UHR was significantly higher in patients with metabolic syndrome (14.76 ± 6.33%) compared with those without metabolic syndrome (10.0 ± 3.10%) (*p* < .001). People with high UHR are 2.9 times more at risk of metabolic syndrome and the best cut‐off point was 9.50% with 86% sensitivity and 55% specificity.

**Conclusions:**

According to our study, UHR is also helpful in diagnosing metabolic syndrome and can also be used to screen people at risk for metabolic syndrome.

## Graphical abstract

This cross‐sectional study determines a parameter to more easily diagnose metabolic syndrome. Data related to 96 people with metabolic syndrome and 721 people without metabolic syndrome were analyzed. The mean UHR was significantly higher in patients with metabolic syndrome compared with those without metabolic syndrome (P < .001). People with high UHR are 2.9 times more at risk of metabolic syndrome, and the best cut‐off point was 9.50% with 86% sensitivity and 55% specificity.

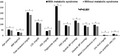

## 1 BACKGROUND AND OBJECTIVES

Metabolic syndrome refers to the existence of a set of risk factors for cardiovascular disease.^1^ People with metabolic syndrome are at a higher risk of developing cardiovascular disease and diabetes than others. Some of these influencing factors include dyslipidaemia, central or visceral obesity and insulin resistance.^2^, ^3^, ^4^

Recent epidemiological studies have shown that the risk factors of metabolic syndrome cannot justify all cardiovascular events.^4^ In recent studies, in addition to these factors, other factors such as inflammatory markers, microalbuminuria and hyperuricaemia have been suggested to justify these events. Some of these studies have suggested that insulin resistance is the underlying cause of metabolic syndrome. Increased insulin secretion may increase blood uric acid levels due to its role in reducing renal excretion of uric acid and sodium, thereby affecting cardiovascular events.^3^, ^5^ Studies have also shown that high serum uric acid levels increase the incidence and mortality of cardiovascular disease compared to normal levels.^6^, ^7^, ^8^

Uric acid is a product of purine catabolism that causes insulin resistance and increasing atherosclerosis by reducing the production of nitric oxide, the proliferation of vascular smooth muscle and endothelial dysfunction.^4^ According to a study by Duman et al.^9^, serum uric acid levels in patients with poor control diabetes are significantly higher than in patients with well‐controlled diabetes and there is a strong significant correlation between serum uric acid levels and HbA1c. In addition, elevated uric acid levels may indicate a cellular level of damage and serve as a prognostic marker in patients with pre‐diabetes and type 2 diabetes.^10^ Therefore, serum uric acid level can be considered as a prognostic parameter in patients with metabolic syndrome.

Recent studies also show a higher prevalence of metabolic syndrome in people with hyperuricaemia.^11^, ^12^, ^13^ Hyperuricaemia is a laboratory disorder that is more common in children, but because the diagnostic value of uric acid alone is low, paediatricians often do not examine its serum level.^14^ The results of some studies indicate a strong relationship between uric acid levels and metabolic syndrome in adults and children and have suggested various mechanisms to justify this relationship.^11^, ^15^

The ratio of uric acid to HDL is a marker that increases in inflammatory conditions.^16^ In Kocak et al.’s (2019)^17^ study, the use of UHR as an effective tool to diagnose metabolic syndrome in patients with type 2 diabetes has been proposed. UHR also used as a marker that is significantly associated with FPG and HbA1c levels to assess the control of type 2 diabetes in men,^18^ hepatic steatosis,^19^ Hashimoto's thyroiditis^16^ and non‐alcoholic fatty liver disease.^20^

Since the prevalence of hyperuricaemia and metabolic syndrome is increasing in many countries and due to the complications of metabolic syndrome, its early diagnosis is very important. Since the studies on the relationship between uric acid and the diagnosis of metabolic syndrome are insufficient, we perform this study to determine a parameter for easier diagnosis of metabolic syndrome and its screening, so we investigated the relationship between the ratio of uric acid to HDL and metabolic syndrome.

## 2 METHODS

### 2.1 Population and study design

The target population in this cross‐sectional study, which was conducted to determine the relationship between serum uric acid levels and the ratio of serum uric acid to HDL (UHR) levels with metabolic syndrome, is the same study in the Kerman Coronary Artery Disease Risk Factor Study (KERCADRS). This study was performed on the urban population of Kerman (the largest city in south‐eastern Iran with a population of about 720,000 people). The protocol for conducting this study has been reviewed and approved by the ethics committee (Ethics Code 88‐110KA).^21^

People with diabetes or taking certain medications such as anticonvulsants and corticosteroids were excluded from the study.

The height of the subjects was measured with a meter having the least count of 0.1 cm in the standing position without shoes and when the shoulders were in the normal resting position. Weight was measured with a shoeless dress with a 707 seca scale accurate to 100 g. The device can measure 0.1–150 kg. Patients’ waist at the umbilical level was measured with a tape while wearing minimal clothing.

Blood pressure with a standard manometer (Model RISHTER, Germany), fasting sugar (KIMIA Kit, Code 890410, Iran), triglyceride (KIMIA Kit, Code 890201, Iran), total cholesterol (KIMIA Kit, Code 890303, Iran) and HDL (HDL ‐ PARS Kit, Code 89022, Iran) were measured, and LDL was measured with Frieldwald formula [LDL = Total cholesterol − (HDL + TG/5)].

Blood pressure measurements were performed for patients after sitting for at least 10 min. If abnormal blood pressure was detected, it was measured again at least 30 min after the initial measurement.

Serum uric acid levels were measured by the colorimetric method. After collecting data, the subjects were divided into two groups with metabolic syndrome and the group without metabolic syndrome.

In this study, metabolic syndrome was defined based on ATP‐III criteria as having three or more of the following criteria:

Abdominal obesity greater than 88 cm for women and 102 cm for men, blood pressure greater than 130/85 mmHg or taking antihypertensive drugs, HDL ≤ 40 mg/dl, triglycerides (TG) ≥150 mg/dl, FBS ≥ 100 mg/dl.^1^

### 2.2 Statistical analysis

Data analysis in the present study was performed using STATA software (version 14.0, Stata Corp, College Station, TX, USA). Qualitative data were reported as numbers and percentages. Considering the large sample size, the distribution of all variables in our study was normal and quantitative data were reported as averages and standard deviations. Comparison between qualitative data between the two groups was done using chi‐squared test and comparison between quantitative variables between the two groups was done using independent t‐test. Univariate and multivariate regression analyses were performed to identify the predictors of metabolic syndrome in patients.

ROC analysis was performed using STATA software (version 14.0, Stata Corp) to determine the sensitivity and specificity of the main variables in predicting metabolic syndrome in patients. The *p* values lower than .05 were considered statistically significant.

## 3 RESULTS

In this study, data related to 817 people, including 96 people with metabolic syndrome and 721 people without metabolic syndrome, were analysed. The basic characteristics of the two groups with and without metabolic syndrome are shown in Table 1. The results of the analysis showed that in the group with metabolic syndrome, 43 (44.79%) were male and 53 (55.21%) were women; in the group without metabolic syndrome, 325 (45.08%) were male and 396 (54.92%) were female, but there was no significant difference between the two groups in terms of gender (*p* = .95).

**TABLE 1:** Comparison of demographic findings baseline and characteristics of individuals in the two groups with and without metabolic syndrome

	With metabolic syndrome	Without metabolic syndrome	*p* Value
Gender			
Male (*n* (%))	43 (44.79)	325 (45.08)	.95
Female (*n* (%))	53 (55.21)	396 (54.92)	

	Mean ± Standard deviation		
Age (years)	53.29 ± 11.57	46.12 ± 14.97	<.001
BMI (kg/m^2^)	30.75 ± 4.07	26.22 ± 4.58	<.001
Total cholesterol (mg/dl)	206.27 ± 51.01	184.65 ± 37.36	<.001
LDL (mg/dl)	115.85 ± 42.49	108.65 ± 32.12	<.001
HDL (mg/dl)	39.92 ± 8.93	49.50 ± 10.45	<.001
Triglyceride	261.68 ± 111.34	133.87 ± 74.57	<.001
FBS (mg/dl)	91.08 ± 12.51	83.48 ± 11.42	<.001
Systolic BP (mmHg)	132.96 ± 19.51	113.70 ± 16.82	<.001
Diastolic BP (mmHg)	89.06 ± 11.69	77.71 ± 10.50	<.001
Waist Circumference (cm)	95.63 ± 8.16	82.83 ± 10.86	<.001
Uric Acid (mg/dl)	5.52 ± 1.44	4.66 ± 1.18	<.001
Uric acid/HDL ratio (%)	14.76 ± 6.33	10.0 ± 3.10	<.001

Abbreviations: BMI, Body mass index; BP, Blood pressure; FBS, Fasting blood sugar; HDL, High‐density lipoprotein; LDL, Low‐density lipoprotein.

Accordingly, the mean age in the group with metabolic syndrome was 53.29 ± 11.57 years and in the group without metabolic syndrome was 46.12 ± 14.97 years which there was a significant difference between the two groups (*p* < .001).

Similarly, there was a significant difference between the two groups in terms of BMI, total cholesterol, LDL, HDL, triglyceride, fasting sugar, systolic and diastolic blood pressure and waist circumference (*p* < .001). The mean uric acid level in people with metabolic syndrome and without metabolic syndrome was 5.52 ± 1.44 and 4.66 ± 1.18 mg/dl respectively (*p* < .001). The mean ratio of uric acid to HDL in people with metabolic syndrome and without metabolic syndrome was 14.76 ± 6.33 and 10.0 ± 3.10% respectively (*p* < .001). There was a significant difference in these parameters between the two groups.

Univariate analysis was performed to calculate the odds ratio of metabolic syndrome if the level of any of the above parameters is not normal without considering other parameters. The results of univariate analysis are shown in Table 2; accordingly, patients with low HDL (≤40 mg/dl), high triglycerides (≥110 mg/dl), fasting blood sugar above normal (≥100 mg/dl), high systolic (≥130 mmHg) and diastolic blood pressure (≥85 mmHg), high waist circumference, hyperuricaemia and high ratio of uric acid to HDL had a significantly higher risk of developing metabolic syndrome.

**TABLE 2:** Univariate regression analysis of variables according to their association with metabolic syndrome

Variable	Metabolic syndrome OR (95% CI)	*p*‐Value
HDL (mg/dl)	4.38 (2.21–8.70)	<.001
Triglyceride (mg/dl)	11.11 (6.97–17.72)	<.001
FBS (mg/dl)	2.25 (1.27–3.98)	<.001
Systolic BP (mmHg)	8.44 (5.33–13.37)	<.001
Diastolic BP (mmHg)	11.04 (6.73–1.12)	<.001
Waist circumference (cm)	12.72 (7.90–20.48)	<.001
Uric acid (mg/dl)	1.48 (1.16–1.89)	<.001
Uric acid/HDL ratio (%)	2.90 (2.12–3.97)	<.001

Abbreviations: BP, Blood pressure; FBS, Fasting blood sugar; HDL, High‐density lipoprotein.

To evaluate the predictability of the above variables, multivariate logistic regression analysis was performed by the Backward Elimination method; the results of this analysis showed that uric acid alone is not a predictor of metabolic syndrome, so it was excluded from the final model. According to this analysis, in interaction with other variables, people with low HDL were 2.98 times and people with high uric acid to HDL ratio were 1.84 times more at risk of metabolic syndrome. The results related to the multivariate logistic regression analysis are given in Table 3.

**TABLE 3:** Independent associations with metabolic syndrome using multivariate regression analysis

Variable	Metabolic syndrome OR (95% CI)	*p*‐Value
HDL (mg/dl)	2.89 (1.20–6.94)	.01
Triglyceride (mg/dl)	10.92 (5.71–20.88)	<.001
Systolic BP (mmHg)	2.42 (1.24–4.71)	<.001
Diastolic BP (mmHg)	12.66 (5.85–27.38)	<.001
Waist Circumference (cm)	12.72 (7.90–20.48)	<.001
Uric Acid/HDL ratio (%)	1.84 (1.27–2.66)	.001

Abbreviations: BP, Blood pressure; HDL, High‐density lipoprotein.

**FIGURE 1:**
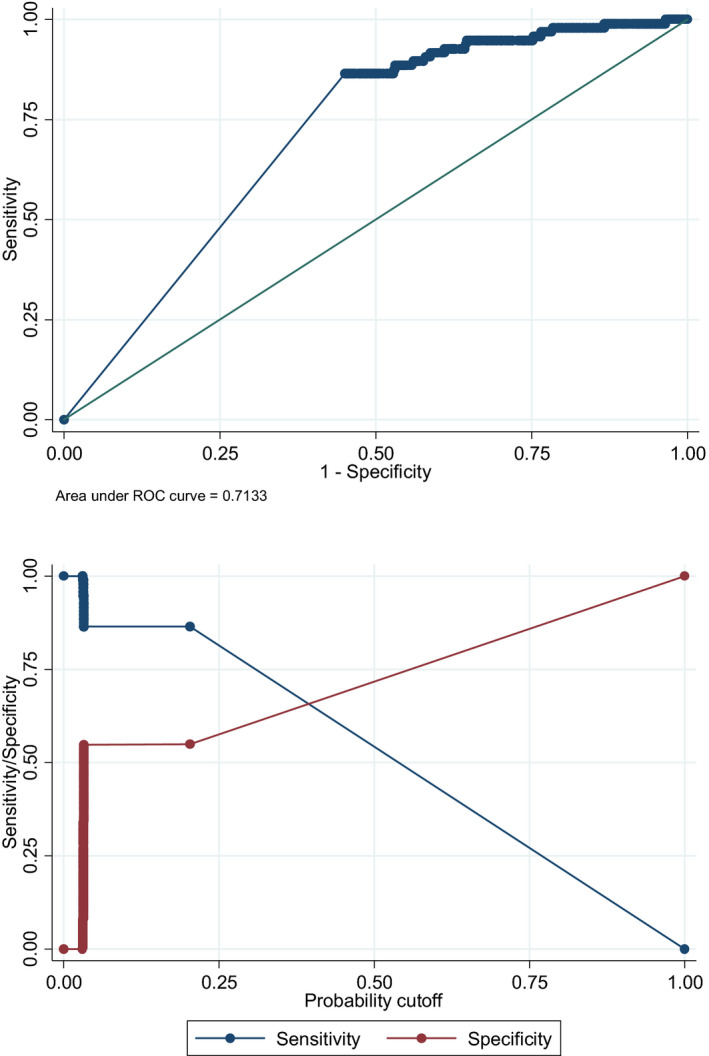
Receiver operating characteristic (ROC) curve of the sensitivity and specificity of uric acid to HDL ratio in the diagnosis of metabolic syndrome

ROC analysis was performed to determine the sensitivity and specificity of uric acid to HDL and set a cut‐off point for the diagnosis of metabolic syndrome. The best cut‐off point for this ratio was 9.50% with 86% sensitivity and 55% specificity (AUC = 0.71) (Figure 1).

## 4 DISCUSSION

According to previous studies, serum uric acid levels are generally higher in men than women, but after 50 years of age, uric acid levels also increase in women.^22^ In the present study, the prevalence of metabolic syndrome was similar in both genders. In another study, no statistically significant association was found between gender and metabolic syndrome, which confirms the results of our study.^23^

The relationship between metabolic syndrome and serum uric acid levels has been well demonstrated in the literature. According to various studies, a higher prevalence of metabolic syndrome has been shown in people with hyperuricaemia.^11^, ^12^, ^13^ A significant association between higher serum uric acid levels and the prevalence of metabolic syndrome showed in previous studies.^24^

In Iran, uric acid is higher in adults with metabolic syndrome, and it seems hyperuricaemia is involved in the prevalence of metabolic syndrome.^25^, ^26^, ^27^ In our study, metabolic syndrome was associated with hyperuricaemia. Another study by Lin et al.^28^ showed that with an abnormal increase in the values of other criteria of metabolic syndrome, the mean serum uric acid level increases. Another study in Japan showed an increased risk of metabolic syndrome following hyperuricaemia.^29^ Some studies also showed that this relationship is stronger in children and adolescents than in the elderly.^15^, ^30^, ^31^ The Sui et al.’s^32^ study showed a relationship between uric acid and metabolic syndrome in both genders.

Elevated serum uric acid levels have often been linked to cardiovascular risk factors such as hypertriglyceridemia, hypertension, obesity and hyperglycaemia, which, if found together in one person, is called metabolic syndrome.^33^, ^34^ The mechanism of association between serum uric acid levels and metabolic syndrome is not well understood, but it appears that when uric acid enters cells through some organic anion carriers, it causes an oxidative process in the vascular smooth muscle, endothelial, fat, islet, renal tubular and liver cells, and therefore, increased serum uric acid increases the risk of metabolic syndrome.^35^ In another study, uric acid was identified as a predictor of metabolic syndrome in older women with newly diagnosed metabolic syndrome.^36^ In our study, it was also shown that without considering other components, uric acid with an odds ratio of 1.48 is associated with metabolic syndrome, but interaction with other components alone is not a good predictor parameter for this syndrome.

Serum levels of uric acid and other metabolic syndrome factors have been evaluated in various studies. In the present study, it was shown that the risk of metabolic syndrome in people with high serum triglyceride levels is 11.11 times higher. In the study of Conen et al.^33^, a close relationship was found between serum triglycerides and serum uric acid levels that can confirm our results.

According to the present study, people with low HDL are more than four times more likely to develop metabolic syndrome than people with normal HDL. And people with a high ratio of uric acid to HDL are 2.9 times more likely to have metabolic syndrome. In the study of Özalp Kızılay et al.^37^, the presence of hyperuricaemia and low HDL is associated with the presence of metabolic syndrome.

In the study of Mansiroglu et al.^38^, uric acid and UHR levels were significantly higher in patients with coronary artery fistula than in controls. However, in their study, the relationship between the presence of metabolic syndrome with UHR levels and coronary artery fistula was not evaluated.

In a retrospective study by Kocak et al., more than 10.6% of UHR had a sensitivity of 83% and specificity of 71% for the prediction of metabolic syndrome in 100 patients with type 2 diabetes.^17^ Although our study was performed on non‐diabetic patients, its results indicated an appropriate sensitivity and specificity for UHR in the diagnosis of patients with metabolic syndrome.

According to our study in non‐diabetics, this ratio is associated with metabolic syndrome, but the chance of developing it is lower than other components, so it is expected due to the higher prevalence of hyperuricaemia and low HDL in diabetics compared to nondiabetic individuals. The ratio of uric acid to HDL in diabetics has a stronger relationship with metabolic syndrome. Considering that the prevalence of diabetes and metabolic syndrome in our country and many countries is increasing and a lot of annual costs and manpower to address complications are caused by them and many of these complications, unfortunately, have detrimental effects on the quality of personal and social life of patients, so study and try to find a parameter that can help in early screening of people at risk is very important. It is hoped that by conducting further studies in these two groups, this component may be introduced as one of the predictors of future metabolic syndrome.

One of the limitations of this study was its sample size, although it was sufficient, studies with a larger sample size could help to more accurately determine the association between UHR and metabolic syndrome and to identify other factors for early detection of metabolic syndrome. Non‐diabetic patients enrolled in the study, and this is one of the strengths of our study. We suggest that further studies be performed on the general population as well as individuals with and without any of the metabolic syndrome factors (e.g., diabetes in our study) that can affect the serum levels of uric acid.

## 5 CONCLUSION

According to our study, there is an association between higher UHR levels and metabolic syndrome, and UHR is helpful in diagnosing metabolic syndrome and can also be used to screen people at risk for metabolic syndrome. However, more studies in larger populations, especially with more attention to people with diabetes, can provide stronger scientific evidence.

## CONFLICT OF INTEREST

None.

## AUTHOR CONTRIBUTION

**Farzaneh Yazdi:** Conceptualization (lead); Investigation (equal); Writing‐review & editing (equal). **Mohammad Hassan Baghaei:** Conceptualization (equal); Investigation (equal); Writing‐original draft (equal); Writing‐review & editing (equal). **Amir Baniasad:** Investigation (equal); Writing‐original draft (lead); Writing‐review & editing (equal). **Ahmad Naghibzadeh‐Tahami:** Formal analysis (lead); Writing‐review & editing (equal). **Hamid Najafipour:** Conceptualization (equal); Investigation (equal); Writing‐review & editing (equal). **Mohammad Hossein Gozashti:** Conceptualization (lead); Investigation (equal); Writing‐review & editing (lead).

## Data Availability

The data that support the findings of this study are available from the corresponding author upon reasonable request.
